# Unusual Presentation of Pneumocephalus With Late Onset During Labour Epidural Analgesia

**DOI:** 10.7759/cureus.39888

**Published:** 2023-06-02

**Authors:** Maria Riga, Evgenia Koursoumi, Georgia G Kostopanagiotou, Paraskevi Matsota

**Affiliations:** 1 2nd Department of Anesthesiology, Attikon University Hospital, Athens, GRC; 2 School of Medicine, National and Kapodistrian University of Athens, Athens, GRC; 3 Anesthesiology, National and Kapodistrian University of Athens, Athens, GRC

**Keywords:** labour, pneumocephalus, epidural analgesia, dural puncture, babinski

## Abstract

A 36-year-old woman with no significant medical history was in active labour and requested labour analgesia. While the epidural technique was performed at the L4-L5 interspace, using the loss of resistance to air technique (LORA), inadvertent dural puncture occurred. Since the patient reported no headache or discomfort, the same procedure was repeated at the L3-L4 interspace successfully. Loss of resistance was reported at 3 cm and the epidural catheter was advanced uneventfully at 8 cm. Aspiration was negative for blood or cerebrospinal fluid (CSF) and a test dose of 2 ml lidocaine 2% was administered epidurally. Within five minutes the patient exhibited a mild hypotensive episode successfully treated with 2.5 mg ephedrine IV, a sensory blockade up to T6 level, and a motor blockade up to T10 level. Both the woman’s and the baby’s vital signs remained stable, no further drugs were administered epidurally and labour progressed painlessly and uncomplicated for 90 minutes with subsequent vaginal delivery of a healthy newborn. During the episiotomy incision repair, the patient complained of light dizziness and nausea. Her vital signs and the arterial blood gases (ABGs) ordered were within normal range, but the neurological examination revealed an isolated Babinski on the right foot. The head CT scan requested indicated a considerable quantity of air within the subarachnoid region. The patient was treated conservatively; symptoms showed steady improvement with total resolution on the sixth day, and the woman was discharged.

This case reemphasizes the possibility of pneumocephalus, which may, in reality, occur more frequently than is commonly recognized without a CT confirmation.

## Introduction

Pneumocephalus is a relatively rare but well-described complication of unintentional dural puncture, especially when the loss of resistance to air technique (LORA) is used to identify the epidural space [1,2]. Despite its diverse symptoms, pneumocephalus is most commonly presented as severe headache with a sudden onset shortly after the introduction of air into the brain cavity [1]. We report a case of a healthy parturient undergoing labour epidural analgesia, who developed the unusual presentation of a headache-free pneumocephalus with late onset following an inadvertent dural puncture.

## Case presentation

A 36-year-old female G2P1, 164 cm tall and weighing 73 kg, was in active labour (5-6 cm of cervical dilatation) and desired labour analgesia. The patient had no known drug allergies or comorbidities (ASA II). Her medical history stated only a head injury due to a car accident, which recovered uneventfully with no chronic headaches or neurological symptoms. She had also received uncomplicated epidural labour analgesia six years ago.

After consent had been obtained, epidural insertion was performed under strict aseptic conditions, with monitors in place (heart rate (HR) 80 bpm, blood pressure (BP) 129/68 mmHg and oxygen saturation (SPO_2_) 100%, as well as continuous cardiotocography) and with the patient in the sitting position. In details, 1% lidocaine was injected at the L4-L5 interspace as skin infiltration and a Tuohy 18G epidural catheter was inserted in the midline using the LORA (1 ml air) for the identification of the epidural space. However, during the advancement of the epidural needle, an inadvertent dural puncture occurred, confirmed by cerebrospinal fluid (CSF) leakage. The patient reported no headache or discomfort of any kind, so the same procedure was repeated at the L3-L4 interspace successfully. The epidural space was identified using the LORA technique (1 ml air) at 3 cm, and the epidural catheter was advanced uneventfully and left at 8 cm. Blood and CSF aspiration from the epidural catheter were both negative, and 2 ml of lidocaine 2% with epinephrine 1:2,00,000 was administered epidurally as a test dose. Approximately five minutes after administration, a sensory blockade up to T6 level and a motor blockade up to T10 level were reported, which lasted approximately 40 minutes. The patient exhibited only a mild hypotensive episode (BP: 101/67 mmHg), which was successfully treated with 2.5mg ephedrine IV. The patient was reassured, and no further drugs were administered via the epidural catheter, with the assumption of subarachnoid block due to the dural puncture. Both the woman’s and the baby’s vital signs remained stable. Thereafter, labour progressed painlessly and uncomplicatedly for 90 minutes, with the subsequent vaginal delivery of a newborn with APGAR scores of 9 and 10 after one and five minutes, respectively.

**Figure 1:**
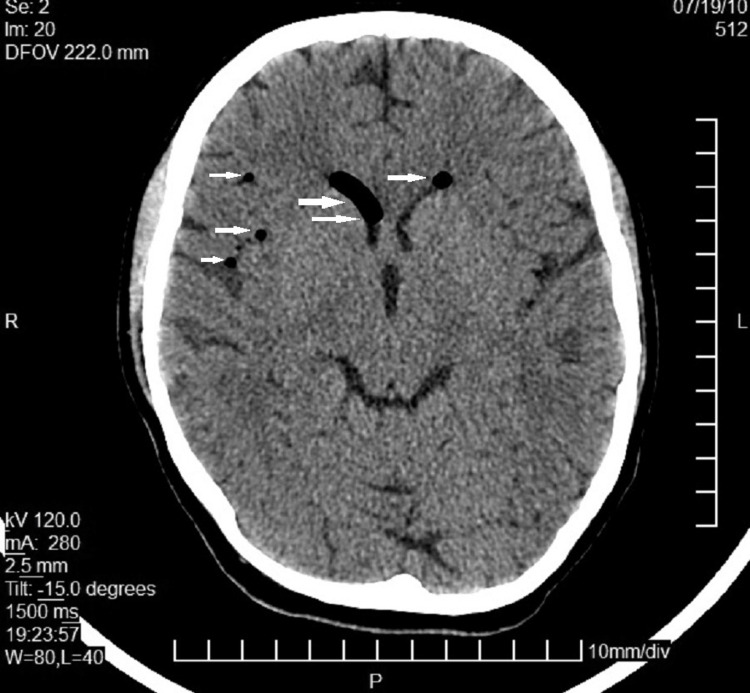
CT day 1 Presence of air intracranially (white arrows)

A few minutes after the vaginal delivery and during the episiotomy incision repair, the patient complained of light dizziness associated with nausea. Both her vital signs and the arterial blood gases (ABGs) ordered were within normal range. The symptoms, however, were sustained, and for that reason, the anesthesiologist in charge conducted a brief neurological assessment (mostly motor and sensory exam), which revealed a Babinski sign on the right foot. A neurology consultation was then ordered, which confirmed no cognitive, motor, sensory, or cranial nerve deficits on examination but the isolated finding of Babinski on the right foot, which led to a head CT scan order. The head CT scan indicated a significant quantity of air in the subarachnoid region, particularly in the ventricles (Figure 1). The patient was once again reassured and treated conservatively with bed rest, oxygen, intravenous and oral fluids, and oral analgesics.

**Figure 2:**
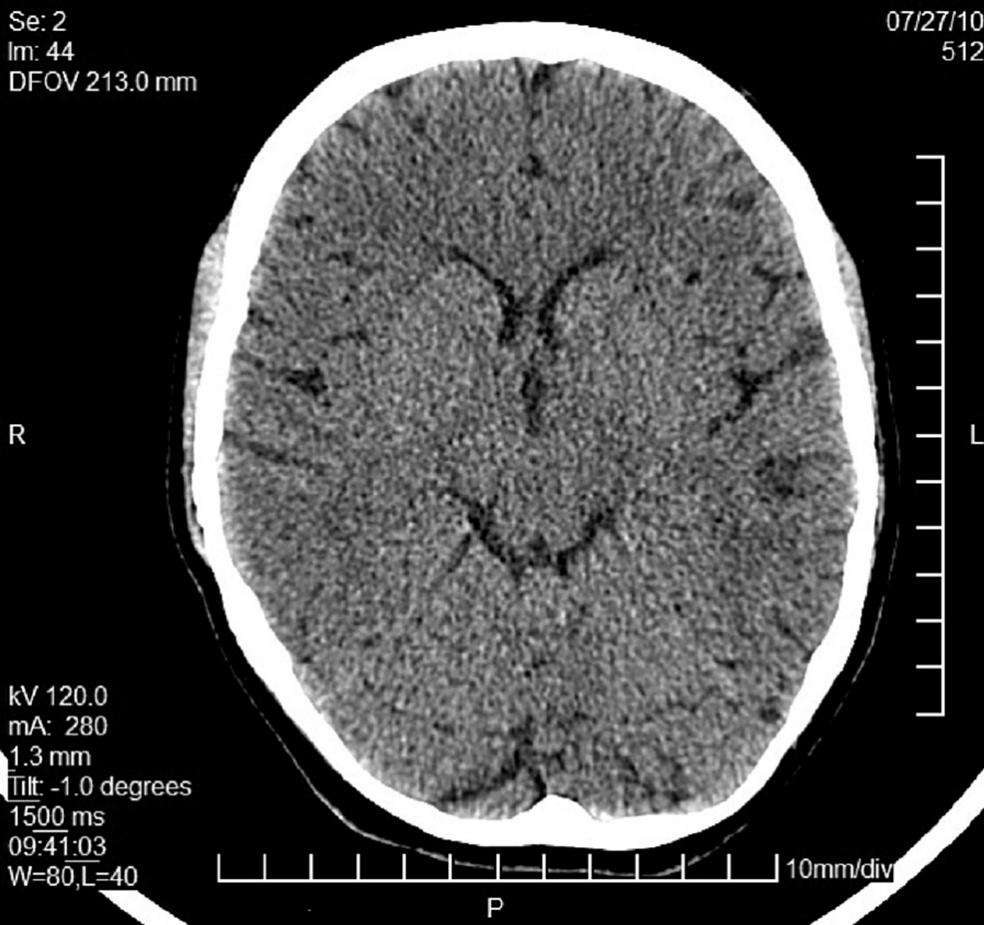
CT day 8 Total reabsorption of air

She remained in the hospital for six days after delivery and was regularly examined by the neurologist and anesthesiologist. All symptoms showed steady improvement, and she was ambulated with no restrictions or further complaints of nausea or dizziness. Two days after the labour, the neurological examination showed clear improvement, still with a neutral Babinski response (no response to stimulation, no plantar flexion or extension). She never developed the typical manifestation of post-dural puncture headache (PDPH), with only a single report of a minor, not orthostatic headache of short duration (one-two hours) at about 48 hours postpartum. On the sixth day, the neurological examination was normal, the woman was discharged, and a second head CT was scheduled in two days, which also revealed total absorption and no air in the subarachnoid space (Figure 2).

## Discussion

Lumbar epidural analgesia has become a standard method of pain relief during labour and delivery throughout the world. LORA or loss of resistance to saline (LORS) is used to identify the epidural space. Although the LORA procedure has been linked to the occurrence of pneumocephalus and intense headaches, prompting some to criticize it, it is not prohibited or completely abandoned [2]. In fact, despite the criticism, there is still no consensus and the LORA technique is often, as in our case, used by many anesthesiologists [1-3].

Pneumocephalus is defined as an intracranial air collection in the extradural, subdural, subarachnoid, intraventricular, or intracerebral compartment [1,4]. Although it is more commonly encountered in the setting of neurosurgical procedures or craniofacial trauma, it can also be encountered after other invasive procedures, including lumbar puncture and spinal or epidural anaesthesia [1,4-6]. It is a relatively rare but well-described complication of unintentional dural puncture during epidural anaesthesia [1]. In that case, pneumocephalus is thought to be induced by the cranial migration of air injected during the LORA technique to identify the epidural space. The cerebral air bubbles act as a space-occupying lesion, irritating the meninges. The subdural or subarachnoid space may serve as an entrance point into the brain. Because the pressure differential favours passage in this direction, subdural air moves quickly to the head, especially when the patient is in the sitting position [3].

The introduction of air into the spinal canal, followed by migration to the cranial cavity, might result in severe neurological symptoms and findings after a physical examination. Severe headache with a typically sudden onset is the most common symptom, along with visual or auditory abnormalities, lethargy, paresthesias, or nausea with vomiting [1]. Physical examination can reveal meningeal symptoms or localised neurologic abnormalities such as hemiparesis, hemiplegia, or cranial nerve palsies [7]. In the most extreme of presentations, patients may experience loss of consciousness with loss of gag reflex and fixed pupils. More rarely, cardiovascular instability has been described, with an incidence of cardiac arrest also reported [8].

Pneumocephalus symptoms usually begin shortly after an inadvertent dural puncture or when air is directly injected into the subdural region following loss of resistance [1]. Although delayed exaggeration or reappearance of symptoms has been reported in the literature, the total absence of any symptom shortly after the dural puncture and the introduction of air is extremely rare [1,9,10].

Despite its dramatic appearance, there have been no known deaths as a result of pneumocephalus [11]. The precise neurological symptoms caused by the presence of air in the cranium are determined by the intracranial distribution of air bubbles, while the length and severity of the symptoms are determined by the volume of air present in the skull [2]. There is no established safe volume of air that can be injected during the LORA procedure. Larger quantities appear to be related with more complications than small volumes, despite the fact that as little as 2-3 ml has been demonstrated to produce pneumocephalus [12,13]. Supine posture, intensive hydration, caffeine, analgesics, and oxygen treatment are all suggested for symptom relief [12]. As the air is reabsorbed, the headache gradually improves over the next four-five days [3,7,13]. However, definitive resolution of symptoms is usually associated with complete reabsorption of intracranial air, which is rather slow and can take up to seven days [14].

In our case, apart from the inadvertent dural puncture and the identification of the epidural space using the LORA procedure, pneumocephalus could not be suspected by the other clinical features. First of all, contrary to the literature, our patient reported no discomfort at all shortly after the dural puncture and the introduction of air, but the symptoms (nausea and dizziness) occurred approximately two hours later. Also, the parturient was hemodynamically stable throughout the whole procedure, apart from the slight drop in BP observed immediately after the administration of the test dose, which was easily managed with a single small dose of ephedrine and is most likely attributable to the subarachnoid block. Most importantly, the manifestation of pneumocephalus was quite unusual in our case. Headache, the most common symptom described in literature, was totally absent, and the patient reported only presyncopic symptoms like dizziness and nausea without a cardiovascular component. Since the symptoms were atypical and transient, it was only the positive Babinski that forced us to further investigate with a CT scan, which revealed the pneumocephalus. The delayed onset of this atypical presentation of pneumocephalus after childbirth observed in our case can be attributed to a late migration of air through the subarachnoid space, which might have been augmented by the increased intra-abdominal pressure during maternal pushing and baby delivery, given that the second performed epidural technique unintentionally resulted in spinal anaesthesia.

## Conclusions

To conclude, this case report reemphasizes the possibility of pneumocephalus following epidural analgesia and dural puncture with the LORA technique. This complication may in reality occur more frequently than is commonly recognised, since its symptoms may be minor or atypical and therefore ignored or easily attributed to the dural puncture and the subarachnoid block in the absence of a CT confirmation.
